# Effects of Different Crude Protein and Dietary Fiber Levels on the Comparative Energy and Nutrient Utilization in Sows and Growing Pigs

**DOI:** 10.3390/ani10030495

**Published:** 2020-03-16

**Authors:** Wenxuan Dong, Gang Zhang, Zhongchao Li, Ling Liu, Shuai Zhang, Defa Li

**Affiliations:** State Key Laboratory of Animal Nutrition, College of Animal Science and Technology, China Agricultural University, Beijing 100193, China; dongwx@cau.edu.cn (W.D.); 18729564631@163.com (G.Z.); lizhongchao2012@163.com (Z.L.) lingliu321@126.com (L.L.)

**Keywords:** adult sows, apparent total tract digestibility, digestible energy, growing pigs, metabolizable energy

## Abstract

**Simple Summary:**

Accurate evaluation of the nutritional values of ingredients fed to sows is highly valuable in swine production, and those values should be kept updated with the genetic improvement of sows. In the feedstuff tables of NRC (2012), the same available energy value was assigned for growing pigs and adult sows for the same ingredient, whereas two different values were used in the feedstuff tables published in China and France (INRA). More research and efforts are required to solve these conflicts, while data gained from animal trials are limited currently. Therefore, we determined and compared the nutritional values of eight ingredients fed to both growing pigs and adult sows, and found that sows had lower available energy and nutrient digestibility when fed soybean meal or cottonseed meal compared with growing pigs, and the crude protein content is a good predictor to estimate the available energy values of ingredients fed to sows based on the values measured from growing pigs. The results of the current study can facilitate the accurate formulation of sow diets.

**Abstract:**

This study was conducted to determine and compare digestible energy (DE) and metabolizable energy (ME) values and the apparent total tract digestibility (ATTD) of energy and nutrients in eight ingredients fed to both growing pigs and sows. Two experiments with 48 crossbred barrows or six non-pregnant sows were allotted to eight treatments in a completely randomized design or a pseudo Latin square with six replicated pigs per dietary treatment. The dietary treatments were formulated with two cereal ingredients: corn and wheat; two ingredients with a high protein level and a low fiber level (HPLF): soybean meal (SBM) and cottonseed meal (CSM); two ingredients with medium protein level and medium fiber level (MPMF): corn distiller’ dried grains with solubles (DDGS) and corn germ meal (CGM); and two ingredients with a low protein level and a high fiber level (LPHF): wheat bran (WB) and palm kernel meal (PKM), respectively. Adult sows had greater DE and ME values and ATTD of energy and nutrients when fed cereal ingredients compared with growing pigs, and had lower DE and ME contents and ATTD of energy and nutrients except for acid detergent fiber (ADF) when fed HPLF ingredients compared with growing pigs. Moreover, no differences were observed between adult sows and growing pigs in DE and ME contents and ATTD of energy and nutrients when fed MPMF and LPHF ingredients, except that adult sows showed a greater ATTD of crude protein (CP) when fed MPMF ingredients compared with growing pigs. Our results indicate that sows had a lower available energy and nutrient digestibility when fed SBM or CSM compared with growing pigs. Crude protein contents in ingredients should be considered when predicting DE and ME values in sows based on the DE and ME values measured from growing pigs.

## 1. Introduction

Adult sows are believed to have greater digestible energy (DE) and metabolizable energy (ME) contents and greater apparent total tract digestibility (ATTD) of nutrients compared with growing pigs (Fermandez et al., 1986; Cozannet et al., 2010; Casas and Stein, 2017) [1,2,3]. However, several studies in the literature also showed that the ME values of some diets fed to sows were not different from or even lower than those fed to growing pigs (Le Goff and Noblet, 2001; Lowell et al., 2015) [4,5]. The differences in energy and nutrient utilization between adult sows and growing pigs are commonly explained by the greater gastrointestinal development and greater capacity for fiber degradation in hindgut in sows (Fermandez et al., 1986; Casas and Stein, 2017) [1,3]. However, chemical compositions of the ingredients, especially protein and fiber, could also influence the available energy and nutrient digestibility of the diets. For instance, excessive dietary crude protein (CP) intake could reduce the energy utilization and feed efficiency in growing pigs, and increase the energy lost through urine and heat in adult sows (Pedersen et al., 2018) [6]. Moreover, dietary fiber (DF) is negatively correlated with the digestibility of nutrients and energy in sows and growing pigs (Le Gall et al., 2009) [7]. Thus, we hypothesized that the CP and DF contents in diets may have influence when comparing the energy and nutrient utilization between sows and growing pigs.

In the feedstuff table of NRC (2012) (NRC, 2012) [8], the ingredient used in swine diets was assigned the same energy value for growing pigs and adult sows whereas separate energy values for sows and growing pigs have been recommended by researchers in the USA recently (Lowell et al., 2015; Casas and Stein, 2017) [3,5]. In the feedstuff tables published in China (National standard for nutrient requirement of swine in China, 2020, in press) and France (INRA) [9], two different available energy values were commonly assigned for growing pigs and sows for the same ingredient. Thus, accurate evaluation of the nutritional values of ingredients fed to sows is highly valuable in swine production. Although predicting the energy values for ingredients used in sows have been proposed from values measured in growing pigs (Le Goff and Noblet, 2001; Lowell et al., 2015) [4,5], those data should be kept updated because of the greater energy intake and body mobilization in modern high-yielding sows nursing 13 to 14 piglets due to the genetic improvement, resulting in the changes in energy and nutrient utilization (Theil et al., 2004; Strathe et al., 2017) [10,11]. However, the research data gained from animal trials are limited currently.

Therefore, the objective of this study was to determine and compare the DE and ME contents and the ATTD of energy and nutrients in eight ingredients with different CP and DF levels fed to both growing pigs and adult sows.

## 2. Materials and Methods

All the protocols used in the experiments were reviewed and approved by the Institutional Animal Care and Use Committee of China Agricultural University (Beijing, China). The animal trials were performed at the Metabolism Laboratory of FengNing Swine Research Unit of China Agricultural University (Academician Workstation in Chengdejiuyun Agricultural & Livestock Co., Ltd, Chengde, China) from May to September, 2019. 

### 2.1. Diets, Animals, and Experimental Design

Eight feed samples including 2 cereal ingredients: corn and wheat, 2 ingredients with a high protein level and a low fiber level (HPLF): soybean meal (SBM) and cottonseed meal (CSM), 2 ingredients with a medium protein level and a medium fiber level (MPMF): corn distiller’ dried grains with solubles (DDGS) and corn germ meal (CGM), and 2 ingredients with low protein level and high fiber level (LPHF): wheat bran (WB) and palm kernel meal (PKM) were collected and analyzed for chemical compositions and gross energy (GE) contents (Table 1). Growing pigs and adult sows were allotted to 8 dietary treatments with different types of premixes and particle sizes but the same ingredient compositions (Table 2). The amount of vitamin and mineral premix was adjusted to meet or exceed the nutrient requirements of growing pigs or adult sows (NRC, 2012) [8], respectively. All ingredients were ground through a 2.0-mm screen for growing pigs and 3.0-mm screen for adult sows. Two diets were formulated to contain 97.0% corn or wheat as the only energy source. The other six ingredients were formulated to replace 30% of corn in the corn diet by the test ingredient, resulting in a final inclusion level of 29.1% for the test ingredient.

In Experiment 1, 48 crossbred growing barrows (Duroc × Large White × Landrace, with initial body weight of 36.5 ± 4.1 kg) were allotted to 8 treatment diets in a completely randomized design with 6 replicated pigs per dietary treatment. The animal trial lasted 22 d, including 7 d for metabolism crates adaptation, 10 d for experimental diet adaptation and 5 d for total feces and urine collection. Fecal collection and urine collection were initiated at 1600 h and 1400 h on d 17, respectively, and ceased at 1600 h and 1400 h on d 22, respectively (i.e., time-based collection method). All pigs were individually housed in stainless-steel metabolism crates (1.4 × 0.7 × 0.6 m) that were equipped with a nipple drinker and a feeder and placed in a temperature-controlled room with the temperature maintained at 22 ± 2 °C. A daily feed allowance equivalent to 4% of the body weight determined at the beginning of animal trial was divided into 2 equal sized meals provided at 0830 and 1530 h, respectively. Water was provided ad libitum via the nipple drinker. The feed refusals and spillages were collected twice daily, dried, and weighed during the 5 d of feces and urine collection period.

In Exp. 2, 6 non-pregnant adult sows (Large White × Landrace, with initial body weight of 208.5 ± 21.7 kg; after parity 2 or 3) were allotted to a pseudo Latin square design with 8 consecutive periods and 8 diets and 6 replicated pigs per dietary treatment. Each period lasted for 15 d, including 10 d for experimental diet adaptation and 5 d for total feces and urine collection. In each collection period, fecal collection and urine collection were initiated at 1600 h and 1400 h on d 0, respectively, and ceased at 1600 h and 1400 h on d 5, respectively (i.e., time-based collection method). In addition, 7 d for metabolism crates adaptation was also included in the whole animal trial. All sows were individually housed in stainless-steel metabolism crates (1.9 × 1.4 × 0.7 m) that were equipped with a nipple drinker and a feeder and placed in a temperature-controlled room with the temperature maintained at 22 ± 2 °C. A daily feed allowance equivalent to 1.5 times ME required for maintenance (MEm) was divided into 2 equal sized meals provided at 0830 and 1530 h, respectively. Water was provided ad libitum via the nipple drinker. The feed refusals and spillages were collected twice daily, dried, and weighed during the 5 d of feces and urine collection period.

### 2.2. Sample Collection

All feces were collected into plastic bags and stored at −20 °C immediately. After each collection period, the total 5-d fecal productions from each pig were pooled and weighed. Approximately 300 g fecal samples were weighed and dried in a forced-air drying oven at 65 °C for 72 h after thawing and mixing thoroughly. Fecal samples were stored at −20 °C for further analysis after drying. Total urine was collected into plastic buckets containing 50 mL of 6 mol/L hydrochloric acid (HCl) and placed under the metabolism crates. The volume of collected urine was measured every day and 10% of the daily urinary collection was stored at −20 °C. Urine samples were pooled for each pig after the collection period. Approximately 45 mL samples were collected for further analysis after thawing and mixing thoroughly.

### 2.3. Chemical Analysis

The samples of ingredients, diets, and feces were ground to pass through 40 mesh screens before analysis and then were analyzed for dry matter (DM; method 930.15), ash (method 942.15), CP (method 990.03), and ether extract (EE; method 920.39) according to AOAC (2007) methods [12]. Filter bags (model F57; Ankom Technology, Macedon, NY, USA) and fiber analyzer equipment (ANKOM200 Fiber Analyzer, Ankom Technology, Macedon, NY, USA) were used to determine the neutral detergent fiber (NDF) and acid detergent fiber (ADF) contents in samples of ingredients, diets, and feces (Van Soest et al., 1991) [13]. Samples of ingredients, diets, feces, and urine were analyzed for GE using an automatic isoperibol oxygen bomb calorimeter (model 6400; Parr1281 Calorimeter, Moline, IL, USA) calibrated by benzoic acid (26.45 MJ/kg). All analyses were conducted in duplicate.

### 2.4. Calculations

Organic matter (OM) was calculated as the difference between DM and ash. The DE and ME values and the ATTD of GE, DM, OM, CP, NDF, and ADF in diets were calculated using the direct method (Adeola, 2001) [14]. The DE and ME values in corn and wheat were calculated as the DE and ME values in the corresponding diets divided by 0.97. The ATTD of energy and nutrients in corn and wheat were considered as the same as those of the corresponding diets. The DE and ME values and the ATTD of GE, DM, OM, CP, NDF, and ADF of the other six ingredients were calculated using the difference method [14] (Adeola, 2001). The difference between the DE or ME values of the same ingredient fed to sows and growing pigs (DEd or MEd) were calculated using the average values of the replicates (data not shown). The calculating equations were described by [14] as follows:

DEf = (GEi − GEf)/DMi(1)

DEfc = DEf/0.97(2)

DEt = (Def − (97.0% − X%) × DEfc)/X%(3)

MEf = (GEi − GEf − GEu)/DMi(4)

MEfc = MEf/0.97(5)

MEt = (MEf − (97.0% − X%) × MEfc)/X%(6)

ATTD = ((Nin − Nout)/Nin) × 100%(7)

ATTDn= (A − B)/F% × 100% + B(8)

The apparent digestible energy (DEf) and metabolizable energy (MEf) content in each diet (feed) (MJ/Kg of DM) can be calculated by Equations (1) and (4), where the total GE intake (GEi) of each pig (MJ/Kg of DM) can be calculated as the result of the GE content of the diet multiplied by DMi. The DMi is the actual total dry matter intake during the collection period; GEf and GEu are the GE content of feces and urine (MJ/Kg of DM) and can be calculated by the product of GE content of the feces or urine multiplied by the dry weight of total feces or the volume of urine recorded over the collection period, respectively. As is shown in Equations (2) and (5), DEfc and MEfc are the corrected apparent digestible and metabolizable energy in the basal diet (MJ/Kg of DM); 0.97 is the percentage of the energy yielding ingredients in this diet. The DE and ME values of the tested ingredients can be obtained using the difference method from Equations (3) and (6), respectively; DEt and MEt are the DE and ME values of the tested ingredients, respectively; X% is the percentage of tested ingredients on diets (29.1%). The ATTD of nutrients in diets can be calculated by Equation (7), where Nin represents the total intake of a certain nutrient in feed, and Nout represents the total fecal output of the homologous nutrient. The ATTD of nutrients (ATTDn) in ingredients can be calculated by Equation (8), where A represents the ATTD of a certain nutrient in the tested diet, and B represents the ATTD of the corresponding nutrient in the basal diet; F% is the percentage of nutrients supplied by ingredients in the test diets.

### 2.5. Statistical Analysis

Data were checked for normality and homogeneity of variances using the UNIVERIATE procedure of SAS 9.2 (SAS Inst. Inc., Carry, NC, USA). Outliers were identified and abandoned in further analysis. Then data were analyzed using the PROC GLM procedure of SAS 9.2 within each of the four categories of ingredients (cereal, HPLF, MPMF, and LPHF). In each category, data from both experiments were combined and subjected to an ANOVA, and the statistical model included the main effects of diet or ingredient (n = 2) and physiological stage (n = 2, sow or growing pig), and the interaction effect between diet or ingredient and stage. The LSMEANS statement was used to calculate the least squares means for each treatment. A preliminary analysis of the data from Experiment 2 indicated that the effect of period and animal were not statistically significant (data not shown). Based on these reasons and also to simplify the statistical analysis, both effects were not considered in the combined analysis (Cozannet et al., 2010) [2]. The correlation coefficients (r) among the chemical compositions and energy values of ingredients were calculated using the CORR procedure of SAS 9.2. Stepwise regression was conducted to select parameters and develop prediction equations for DE and ME values of ingredients fed to sows or growing pigs based on their chemical compositions. The R^2^, *P*-value, Bayesian information criterion (BIC), root mean square error (RMSE) and Akaike’s information criterion (AIC) were used to identify the best-fit equations. In all analyses, the differences were considered significant if *p* < 0.05.

## 3. Results

### 3.1. Effects of Physiological Stage on Cereal Ingredients

There were interactive effects (*p* < 0.05) between physiological stage and ingredient source on ATTD of NDF in cereal grains (Table 3). Specifically, the ATTD of NDF was greater in wheat than corn when fed to growing pigs, but there was no significant difference in ATTD of NDF when cereal grains were fed to adult sows. No interaction between physiological stage and ingredient source was observed on DE and ME contents or the ATTD of GE, DM, OM, CP, and ADF in cereal ingredients. The DE and ME contents and the ATTD of GE, DM, OM, CP, and ADF in cereal grains were greater (*p* < 0.05) when fed to adult sows than fed to growing pigs. Moreover, the ATTD of CP in cereal grains was influenced (*p* < 0.05) by ingredient source, with pigs fed wheat diet having greater ATTD of CP compared with those fed corn diet.

### 3.2. Effects of Physiological Stage on HPLF Ingredients

No interaction between physiological stage and ingredient source was observed on the DE and ME contents or the ATTD of GE, DM, OM, CP, NDF, and ADF in HPLF diets and ingredients (Table 4). The physiological stage had no effects on the DE and ME contents or the ATTD of GE, DM, OM, CP, and NDF in diets containing HPLF ingredients, but the DE and ME contents and the ATTD of GE, DM, OM, CP, and NDF in HPLF ingredients were greater (*p* < 0.05) when fed to growing pigs than fed to adult sows. Adult sows had greater (*p* < 0.05) ATTD of ADF when fed diets containing HPLF ingredients compared with growing pigs. Moreover, pigs fed diets containing SBM showed greater (*p* < 0.01) DE and ME contents and greater (*p* < 0.05) ATTD of GE, DM, OM, CP, NDF, and ADF in both diets and ingredients compared with CSM.

### 3.3. Effects of Physiological Stage on MPMF Ingredients

There were no interactive effects between physiological stage and ingredient source on the DE and ME contents or the ATTD of GE, DM, OM, CP, NDF, and ADF in MPMF diets and ingredients (Table 5). The DE and ME contents and the ATTD of GE, DM, OM, CP, NDF, and ADF in diets containing MPMF ingredients were greater (*p* < 0.05) when fed to adult sows than those fed to growing pigs. Pigs fed diets containing corn DDGS had greater (*p* < 0.05) DE and ME contents in both diets and ingredients compared with CGM.

### 3.4. Effects of Physiological Stage on LPHF Ingredients

There were interactive effects (*p* < 0.05) between physiological stage and ingredient source on the ATTD of DM, OM, NDF, and ADF in LPHF diets and the ATTD of NDF, and ADF in LPHF ingredients (Table 6). Specifically, adult sows had greater ATTD of DM, OM, NDF, and ADF in diets and greater ATTD of NDF and ADF in LPHF ingredients compared with growing pigs when fed diets containing WB; however, there was no significant difference in ATTD of nutrients between adult sows and growing pigs when fed diets containing PKM. The DE and ME contents and the ATTD of GE and CP in diets containing LPHF ingredients were greater (*p* < 0.05) when fed to adult sows than fed to growing pigs. Pigs fed diets containing WB showed greater (*p* < 0.05) ATTD of GE and CP in both diets and ingredients compared with PKM.

### 3.5. Correlation Coefficients and Prediction Equations

As expected, the DE and ME contents in ingredients fed to adult sows and growing pigs were positively correlated with each other (*p* < 0.01, r = 0.85 to 0.96, Table 7). The DE and ME contents in sows were negatively correlated with the ADF contents (*p* < 0.05, r = −0.73; and *p* = 0.05, r = −0.68, respectively) and the ME content in sows was negatively correlated with the ash content (*p* < 0.05, r = −0.72). The DE and ME contents in growing pigs were negatively correlated with the NDF contents (*p* < 0.05, r = −0.77 and −0.73, respectively). A positive correlation was also observed between DEd and MEd (*p* < 0.01, r = 0.98). The CP content showed a negative (*p* < 0.05, r = −0.81) correlation with DEd and a moderately negative correlation (*p* = 0.05, r = −0.69) with MEd. The equations developed to predict DE and ME values in sows based on the DE and ME values measured from growing pigs are presented Table 8. The prediction equations with greater R2 and smaller RMSE, AIC and BIC values were selected. The best-fit equations for DE and ME values in sows (DEs and MEs, respectively) based on the DE and ME values measured from growing pigs (DEg and MEg, respectively) were: DEs = (2.01 × DEg) − (1.03 × MEg) − (0.11 × CP) + 1.23 and MEs = (1.59 × DEg) − (0.59 × MEg) − (0.11 × CP) + 0.73, in which chemical components were expressed in % DM and energy were expressed as MJ/kg DM.

## 4. Discussion

It has been reported that the amount of feed intake in sows and growing pigs did not influence the determination of available energy and ATTD of nutrients in diets and ingredients (Casass and Stein, 2017; Li et al., 2018) [3,15]. In the current study, sows were fed diets containing approximately 1.5 times the ME required for their maintenance, whereas growing pigs were fed diets equivalent to 4% of their body weight, which contains approximately 2.5 times the ME required for their maintenance [8] (NRC, 2012). The above feeding levels were also used in previous studies and close to feeding levels used in the commercial swine production (Le Goff and Noblet, 2001; Lowell et al., 2015) [4,5]. Traditionally, the marker-to-marker total collection method is recommended in digestibility trials due to the variation in passage rate of digesta among pigs (Casas and Stein, 2017) [3]. Fecal collection was initiated when the first color marker appeared in the feces and ceased when the second color marker appeared. However, the color of some ingredients (e.g., PKM) used herein could affect the judgement of marker appearance. As a result, the time-based total collection method can also be used in such trials and has been proved as reliable as the marker-to-marker total collection method [16] (Li et al., 2016). Thus, the time-based collection method was used in the current study.

### 4.1. Effects of Physiological Stage on Diets

Cereal diets had greater DE and ME contents and ATTD of GE in sows than in growing pigs, which is consistent with the previous results (Lowell et al., 2015) [5]. Increased DE and ME contents in cereal grains were also observed in growing-finishing pigs with increased body weight (Xie et al., 2017) [17]. Moreover, diets containing MPMF and LPHF ingredients showed greater available energy in sows compared with those in growing pigs, which is in agreement with the previous studies (Le Goff and Noblet, 2001; Lowell et al., 2015) [4,5]. In addition, sows also had greater ATTD of DM, OM, CP, NDF, and ADF compared with growing pigs when fed diets containing cereal grains, MPMF ingredients, and LPHF ingredients excluding PKM. Since feeding levels did not affect the determination of available energy and nutrient digestibility in diets or ingredients (Casas and Stein, 2017; Li et al., 2018) [3,15], the increased dietary DE and ME contents and nutrients digestibility in sows could be explained by their greater body weight [17,18] (Noblet and Van Milgen, 2004; Xie et al., 2017). With greater body weight, adult sows have larger intestinal volume, in which digesta could be more exposed to the digestive enzymes and hindgut microbiota fermentation. As a result, adult sows showed a greater digestive capacity compared with growing pigs [3,5] (Lowell et al., 2015; Casas and Stein, 2017). Therefore, sows had greater energy and nutrient utilization in diets containing cereal, MPMF, and LPHF ingredients than growing pigs. Similarly, weaning pigs (600 μm) and growing pigs (600 μm, the particle size used in the current study) require finer feed particle size to reach the optimum nutrient digestibility than gestating sows (800 μm, the particle size used in the current study) that have greater gastrointestinal digestive capacity (Li et al., 2018) [19].

In agreement with the current study, Lowell et al. (2015) reported that the ME values in sows were not different from those in growing pigs fed the SBM, canola meal (CM), conventional DDGS, and low-fat DDGS diets [5]. When the protein to energy ratio in diets is unbalanced, energy loss in urine and heat are likely to increase in adult sows, and body mobilization should be considered when evaluating the feed efficiency of sows [6] (Pedersen et al., 2018). A reduced protein diet improves energy efficiency and mitigate heat production associated with lactation in sows (Zhang et al. 2020) [20]. In the current study, dietary protein was under-supplied in all diets except for the HPLF diets (NRC, 2012) [8]. The effects of body weight (positive) and over-supplied dietary protein (negative) in HPLF diets were confounded, and it is not surprising that the DE and ME contents and the ATTD of GE, DM, OM, CP, and NDF in sows were not different from those in growing pigs. Unlike the other nutrients, the ATTD of ADF in HPLF diets was greater in sows compared to growing pigs in the current study. The fact that the comparative ATTD of ADF in HPLF diets was different from the digestibility of other nutrients is difficult to explain biologically. We might attribute this discrepancy to the preference for the fermentation substrate by the microbe in the hindgut of sows, or the analytical mistakes in fiber fractions, but more research is needed to further clarify the underlying mechanisms. 

### 4.2. Effects of Physiological Stage on Ingredients

The DE and ME contents in ingredients determined in the current study were close to the expected values (Sauvant et al., 2004; NRC, 2012) [8,9]. Few studies have reported the comparative available energy values and nutrient digestibility of ingredients fed to adult sows and growing pigs. Therefore, no references could be found to verify our results on ingredients.

High levels of dietary fiber could decrease the digestibility of energy and nutrients in sows and growing pigs and have the potential to diminish the digestion efficiency (Le Gall et al., 2009) [7]. This may be because dietary fiber can reduce the exposure time of digesta to enzymes and hindgut microbiota, increase the passage rate of the nutrient flow, and have a significant influence on the large intestinal turnover (Ehle et al., 1982; Serena et al., 2008) [21,22]. Pigs cannot reach their full digestive potential when consuming fiber-rich ingredients, as the digesta would be excreted from the hindgut before it has fully been fermented, especially for adult sows fed a restricted diet. Therefore, physiological stage had no significant influence on the DE and ME contents or the ATTD of GE and nutrients in MPMF ingredients and LPHF ingredients calculated using the difference method, except that the ATTD of CP in MPMF ingredients was greater in sows than growing pigs. We might attribute this discrepancy to the different amino acid profiles among different ingredients, but more reasonable explanations still rely on further studies since amino acid profiles were not analyzed in the current study. 

In HPLF ingredients, we observed significantly greater DE and ME contents and ATTD of GE, DM, OM, CP, and NDF when fed to growing pigs compared with adult sows. Lowell et al. (2015) reported that the DE and ME in diets containing SBM and CM were very close in sows and growing pigs with significantly difference in the basal diet [5]. Although they did not calculate the energy value in ingredients, we may expect the same results as those of the current study. Similarly to the results in diets, the ATTD of ADF in HPLF ingredients were lower in growing pigs compared with those in adult sows, still differ from the comparative tendencies of the other nutrients. This deviation may be introduced by the difference method (Stein et al., 2006; Cozannet et al., 2010; Liu et al., 2013; Li et al., 2017) [2,23,24,25] and greatly rely on the results in diets. 

In addition, a finer particle size was designed in growing pig diets compared with sow diets in the current study just to keep it consistent with the practical production situation, which may have affected the results because reduced particle size could increase the DE and ME values and nutrient digestibility of the ingredients [26]. However, we only observed greater available energy and nutrient digestibility in growing pigs fed the HPLF ingredients compared with sows, indicating that the particle size may not be an influential factor on the results.

Traditionally, in the feedstuff tables commonly used, some feed ingredients were divided into different categories based on one key physiochemical property that would directly affect their nutrient values. For example, wheat, soybean meal, and fish meal were categorized by their crude protein contents, corn DDGS and full-fat rice bran were categorized by the ether extract contents, and sorghum was categorized by its tannin contents (Sauvant et al., 2004; NRC, 2012) [8,9]. Therefore, we think that the prediction of DE and ME values in sows based on the measurements in growing pigs should also be categorized by the different CP contents of ingredients based on the results of the current study. For instance, lower energy value in ingredients with high CP level and greater energy value in ingredients with low CP level should be assigned in sows compared with those in growing pigs, although the range of CP levels need to be further explored.

### 4.3. Correlation Coefficients and Prediction Equations

The DE and ME contents were negatively correlated with the fiber components. Specifically, ADF showed negative correlation with DE and ME contents in sows and NDF was negatively correlated with DE and ME contents in growing pigs. Both NDF and ADF had negative effects in the determination of available energy in feed ingredients fed to sows and growing pigs (Cozannet et al., 2010; Lowell et al., 2015; Pan et al., 2016; Dong et al., 2019) [2,5,27,28,29]. In agreement with our results, it was also reported that the CP contents have a weak correlation with DE and ME values (Cozannet et al., 2010; Lowell et al., 2015; Pan et al., 2016; Dong et al., 2019) [2,5,27,28,29]. Prediction equations for available energy values in sows based on available energy values measured in growing pigs has already been proposed by Le Goff and Noblet (2001) [4]. Therefore, we tried to determine the correlation between chemical compositions and the different available energy values of sows from growing pigs (DEd or MEd). We hypothesized that there were significant correlations between DEd or MEd values and the fiber fraction or CP contents. The results of the current study partially prove our hypothesis that the CP content in ingredients was negatively correlated with DEd and Med, which has never been reported before. However, dietary fiber content did not have significant correlation with DEd and Med, as shown in our study, which might be due to the irregularity between fiber contents and DEd or MEd values among different ingredients. As a result, CP was the only predictor selected from the chemical composition parameters to estimate the DE and ME values in sows (DEs or MEs) using the DE and ME values determined in growing pigs (DEg or MEg). With the addition of CP, the R^2^ of the prediction equations for the DEs and MEs values improved from 0.94 to 0.98 and from 0.96 to 0.97, respectively. Previously, Cozannet et al. (2010) predicted the DEs values in wheat DDGS only based on the physicochemical properties [2] while Lowell et al. (2015) predicted the DEs and MEs values only based on the DEg or MEg values [5]. Moreover, fiber components were included as parameters in predicting the DEs and MEs based on DEg and MEg (Le Goff and Noblet, 2001) [4]. However, contents of fiber components are not easy to analyze and the values measured in different labs may be greatly variable (Stein et al., 2006; Cozannet et al., 2010) [2,23]; thus, such prediction equations proposed based on fiber indicators should be used with caution. Compared with the fiber fractions, CP is much easier and more accurate to analyze and thus, can be a better predictor in estimating DEs and MEs values based on DEg and MEg values. Nevertheless, further comparative mechanisms of protein utilization in sows and growing pigs should be explored to explain the role of CP in such prediction equations, and more digestibility trials are also needed to verify the accuracy of the equations.

## 5. Conclusions

In conclusion, adult sows do not necessarily have greater DE and ME contents or ATTD of energy and nutrients in all kinds of ingredients compared with growing pigs. Sows had lower available energy and nutrient digestibility when fed soybean meal or cottonseed meal compared with growing pigs. Crude protein contents in ingredients should be considered when predicting DE and ME values in sows based on the DE and ME values measured from growing pigs.

## Figures and Tables

**Table 1:** Analyzed chemical compositions of the ingredients ^1,2^ (%, as-fed basis).

Items	GE, MJ/kg	DM	OM	Ash	CP	EE	NDF	ADF
Corn	16.04	86.37	85.24	1.12	6.97	2.91	9.38	2.64
Wheat	16.34	88.75	87.00	1.75	13.39	1.13	11.05	2.87
Soybean Meal	17.58	89.81	83.21	6.60	42.04	1.86	15.74	9.18
Cottonseed Meal	17.68	90.72	84.56	6.16	42.20	1.28	23.89	15.41
Corn DDGS	19.97	89.37	84.93	4.43	28.05	11.16	27.94	9.45
Corn Germ Meal	17.31	91.03	85.43	5.61	24.16	2.40	32.32	9.66
Wheat Bran	16.84	88.95	82.99	5.95	15.44	3.26	44.14	13.04
Palm Kernel Meal	18.27	91.42	87.33	4.09	16.52	6.13	56.46	32.53

^1^ GE, gross energy; DM, dry matter; OM, organic matter; CP, crude protein; EE, ether extract; NDF, neutral detergent fiber; ADF, acid detergent fiber; Corn DDGS, Corn distiller’ dried grains with solubles. ^2^ Data are the mean of duplicate analyses of each ingredient.

**Table 2:** Ingredient components and analyzed chemical compositions of the experimental diets ^1^ (%, as-fed basis).

Items	Experimental Diets							
	Corn	Wheat	SBM	CSM	DDGS	CGM	WB	PKM
Ingredients								
Corn	97.00	-	67.90	67.90	67.90	67.90	67.90	67.90
Wheat	-	97.00	-	-	-	-	-	-
Soybean Meal	-	-	29.10	-	-	-	-	-
Cottonseed Meal	-	-	-	29.10	-	-	-	-
Corn DDGS	-	-	-	-	29.10	-	-	-
Corn Germ Meal	-	-	-	-	-	29.10	-	-
Wheat Bran	-	-	-	-	-	-	29.10	-
Palm Kernel Meal	-	-	-	-	-	-	-	29.10
Dicalcium phosphate	1.35	1.35	1.35	1.35	1.35	1.35	1.35	1.35
Limestone	0.75	0.75	0.75	0.75	0.75	0.75	0.75	0.75
Salt	0.40	0.40	0.40	0.40	0.40	0.40	0.40	0.40
Premix ^2,3^	0.50	0.50	0.50	0.50	0.50	0.50	0.50	0.50
Total	100.00	100.00	100.00	100.00	100.00	100.00	100.00	100.00
Analyzed chemical compositions in growing pig diets ^4^								
Gross energy, MJ/kg	15.54	15.89	15.92	16.01	16.63	15.82	15.75	16.09
Dry matter	86.73	88.77	87.25	87.93	87.89	88.04	86.98	87.65
Organic matter	83.21	84.55	82.28	82.93	83.49	83.06	82.20	83.21
Ash	3.52	4.22	4.94	5.00	4.46	4.98	4.78	4.44
Crude protein	7.24	13.29	16.13	17.62	13.45	11.80	9.58	9.97
Ether extract	2.46	1.36	1.95	1.90	4.33	2.34	2.64	2.70
Neutral detergent fiber	7.89	11.95	9.46	12.31	13.42	17.96	19.93	25.46
Acid detergent fiber	2.21	3.34	5.51	7.93	5.31	5.38	5.90	11.98
Analyzed chemical compositions in sow diets ^4^								
Gross energy, MJ/kg	15.57	15.94	15.92	15.97	16.69	15.94	15.77	16.01
Dry matter	88.89	88.86	87.16	87.84	87.55	88.10	86.97	87.65
Organic matter	83.25	84.61	82.25	82.76	83.15	83.17	81.98	83.05
Ash	3.64	4.25	4.91	5.08	4.40	4.93	4.99	4.59
Crude protein	7.53	13.49	16.57	17.18	12.75	12.51	9.25	9.70
Ether extract	2.17	2.16	1.87	1.78	3.76	2.31	2.46	2.29
Neutral detergent fiber	8.18	11.04	9.80	12.44	14.32	18.54	18.70	24.40
Acid detergent fiber	2.22	3.15	4.06	5.36	4.69	5.68	5.51	12.25

^1^ SBM, soybean meal; CSM, cottonseed meal; Corn DDGS, Corn distiller’ dried grains with solubles; CGM, corn germ meal; WB, wheat bran; PKM, palm kernel meal; ^2^ Premix provided the following quantities per kilogram of complete growing pig diets: vitamin A as retinyl acetate, 5512 IU; vitamin D_3_ as cholecalciferol, 2200 IU; vitamin E as DL-alpha-tocopheryl acetate, 30 IU; vitamin K_3_ as menadione nicotinamide bisulfite, 2.2 mg; vitamin B_12_, 27.6 μg; riboflavin, 4 mg; pantothenic acid as DL-calcium pantothenate, 14 mg; niacin, 30 mg; choline chloride, 400 mg; folacin, 0.7 mg; thiamin as thiamine mononitrate, 1.5 mg; pyridoxine as pyridoxine hydrochloride, 3 mg; biotin, 44 μg; Mn as MnO, 40 mg; Fe as FeSO_4_ H_2_O, 75 mg; Zn as ZnO, 75 mg; Cu as CuSO_4_•5H_2_O, 100 mg; I as KI, 0.3 mg; Se as Na_2_SeO_3_, 0.3 mg; ^3^ Premix provided the following quantities per kilogram of complete sow diets: vitamin A as retinyl acetate, 5512 IU; vitamin D_3_ as cholecalciferol, 2200 IU; vitamin E as DL-alpha-tocopheryl acetate, 64 IU; vitamin K_3_ as menadione nicotinamide bisulfite, 2.2 mg; vitamin B_12_, 27.6 μg; riboflavin, 5.5 mg; pantothenic acid as DL-calcium pantothenate, 13.8 mg; niacin, 30.3 mg; choline chloride, 551 mg; Mn as MnO, 40 mg; Fe as FeSO_4_ H_2_O, 100 mg; Zn as ZnO, 100 mg; Cu as CuSO_4_•5H_2_O, 100 mg; I as KI, 0.3 mg; Se as Na_2_SeO_3_, 0.3 mg; ^4^ The data are the mean of duplicate analyses of each diet.

**Table 3:** Comparative energy and nutrient utilization in cereal ingredients by adult sows and growing pigs ^1^ (dry matter basis).

Items	Corn		Wheat		SEM	*p*-Value		
	Sow	Growing	Sow	Growing		Ingredient	Stage	Interaction
Energy content, MJ/kg								
DE	17.16	16.48	16.99	16.38	0.14	0.368	<0.001	0.811
ME	16.54	15.76	16.32	15.52	0.17	0.193	<0.001	0.975
ME/DE	0.96	0.96	0.96	0.95	0.01	0.076	0.121	0.264
Apparent total tract digestibility, %								
GE	92.39	88.19	92.27	89.44	0.66	0.396	<0.001	0.311
DM	92.52	88.41	91.13	88.75	0.65	0.428	<0.001	0.193
OM	94.45	90.19	93.28	90.54	0.55	0.467	<0.001	0.188
CP	86.88	80.70	93.29	89.36	0.95	<0.001	<0.001	0.250
NDF	75.02 ^a^	48.40 ^c^	72.54 ^a^	57.91 ^b^	2.67	0.216	<0.001	0.042
ADF	68.55	37.38	59.75	40.22	3.63	0.422	<0.001	0.125

^1^ SEM, standard error of the mean; DE, digestible energy; ME, metabolizable energy; GE, gross energy; DM, dry matter; OM, organic matter; CP, crude protein; EE, ether extract; NDF, neutral detergent fiber; ADF, acid detergent fiber; ^a–c^ Means within a row without a common superscript letter differ (*p* < 0.05).

**Table 4:** Comparative energy and nutrient utilization in high protein low fiber (HPLF) diets and ingredients by adult sows and growing pigs ^1^ (dry matter basis).

Items	Soybean Meal		Cottonseed Meal		SEM	*p*-Value		
	Sow	Growing	Sow	Growing		Ingredient	Stage	Interaction
Energy content in diets, MJ/kg								
DE	16.12	16.11	14.71	14.71	0.13	<0.001	0.980	0.975
ME	15.20	15.31	13.94	13.98	0.16	<0.001	0.641	0.833
ME/DE	0.94	0.95	0.95	0.95	0.01	0.668	0.441	0.730
Energy content in ingredients, MJ/kg								
DE	15.47	17.14	11.02	12.70	0.44	<0.001	0.002	0.986
ME	13.77	15.86	9.82	11.67	0.55	<0.001	0.002	0.824
ME/DE	0.89	0.92	0.89	0.92	0.03	0.956	0.261	0.890
Apparent total tract digestibility of diets, %								
GE	88.35	88.20	80.77	80.90	0.73	<0.001	0.989	0.847
DM	88.21	89.13	80.61	80.82	0.78	<0.001	0.484	0.655
OM	91.18	90.69	83.87	83.65	0.65	<0.001	0.593	0.837
CP	87.68	89.15	80.11	80.11	0.92	<0.001	0.714	0.712
NDF	68.22	62.95	40.43	35.31	3.24	<0.001	0.124	0.982
ADF	79.70	65.32	43.11	20.56	3.09	<0.001	<0.001	0.203
Apparent total tract digestibility of ingredients, %								
GE	79.04	87.56	56.52	65.16	2.28	<0.001	0.002	0.979
DM	78.49	90.76	54.05	63.86	2.49	<0.001	<0.001	0.636
OM	83.55	91.84	59.59	68.63	2.12	<0.001	<0.001	0.865
CP	88.00	92.69	77.41	79.87	1.27	<0.001	0.013	0.406
NDF	60.27	83.84	13.78	27.00	6.55	<0.001	0.013	0.449
ADF	85.95	81.07	34.61	14.91	4.47	<0.001	0.015	0.123

^1^ SEM, standard error of the mean; DE, digestible energy; ME, metabolizable energy; GE, gross energy; DM, dry matter; OM, organic matter; CP, crude protein; EE, ether extract; NDF, neutral detergent fiber; ADF, acid detergent fiber.

**Table 5:** Comparative energy and nutrient utilization in medium protein medium fiber (MPMF) diets and ingredients by adult sows and growing pigs ^1^ (dry matter basis).

Items	Corn DDGS		Corn Germ Meal		SEM	*p*-Value		
	Sow	Growing	Sow	Growing		Ingredient	Stage	Interaction
Energy content in diets, MJ/kg								
DE	16.47	15.81	15.43	14.76	0.19	<0.001	0.003	0.986
ME	15.71	14.84	14.52	13.74	0.28	<0.001	0.009	0.881
ME/DE	0.95	0.94	0.94	0.93	0.01	0.338	0.388	0.848
Energy content in ingredients, MJ/kg								
DE	17.20	16.45	13.46	12.97	0.66	<0.001	0.361	0.845
ME	15.99	14.61	11.77	10.97	0.95	<0.001	0.267	0.769
ME/DE	0.93	0.89	0.87	0.85	0.04	0.262	0.493	0.886
Apparent total tract digestibility of diets, %								
GE	87.11	85.87	85.87	81.55	1.24	0.241	<0.001	0.957
DM	86.96	83.06	85.97	82.21	1.05	0.401	0.002	0.947
OM	89.47	85.26	88.31	84.59	0.86	0.307	<0.001	0.778
CP	83.54	78.46	82.12	75.15	1.64	0.167	0.002	0.571
NDF	72.29	53.78	70.81	60.82	2.28	0.239	<0.001	0.079
ADF	74.97	56.50	68.04	59.28	2.81	0.472	<0.001	0.102
Apparent total tract digestibility of ingredients, %								
GE	76.97	73.60	70.77	68.20	3.12	0.092	0.375	0.905
DM	74.36	70.92	71.41	68.42	3.45	0.439	0.365	0.948
OM	78.10	73.97	74.35	71.85	2.89	0.318	0.261	0.779
CP	81.53	77.07	78.80	71.12	2.73	0.131	0.040	0.565
NDF	70.49	59.29	68.42	69.74	3.68	0.269	0.195	0.109
ADF	78.47	66.98	67.76	71.02	4.34	0.453	0.356	0.106

^1^ SEM, standard error of the mean; DE, digestible energy; ME, metabolizable energy; GE, gross energy; DM, dry matter; OM, organic matter; CP, crude protein; EE, ether extract; NDF, neutral detergent fiber; ADF, acid detergent fiber.

**Table 6:** Comparative energy and nutrient utilization in low protein high fiber (LPHF) diets and ingredients by adult sows and growing pigs ^1^ (dry matter basis).

Items	Wheat Bran		Palm Kernel Meal		SEM	*p*-Value		
	Sow	Growing	Sow	Growing		Ingredient	Stage	Interaction
Energy content in diets, MJ/kg								
DE	15.38	14.64	15.07	14.70	0.14	0.365	<0.001	0.176
ME	14.73	14.02	14.30	14.21	0.17	0.479	0.027	0.076
ME/DE	0.96	0.96	0.95	0.97	0.01	0.751	0.353	0.349
Energy content in ingredients, MJ/kg								
DE	12.96	12.23	11.99	12.48	0.43	0.418	0.779	0.179
ME	12.17	11.61	11.32	12.23	0.51	0.742	0.821	0.061
ME/DE	0.94	0.95	0.94	0.98	0.03	0.882	0.272	0.298
Apparent total tract digestibility of diets, %								
GE	84.93	80.70	82.11	80.49	0.71	0.047	<0.001	0.084
DM	84.53 ^a^	79.96 ^b^	87.72 ^a,b^	81.61 ^b^	0.71	0.912	<0.001	0.026
OM	86.98 ^a^	82.66 ^c^	85.27 ^a,b^	84.14 ^b,c^	0.58	0.845	<0.001	0.014
CP	82.83	75.22	72.88	68.37	1.83	<0.001	<0.001	0.410
NDF	63.91 ^b^	48.82 ^c^	72.48 ^a^	67.74 ^a,b^	1.31	<0.001	<0.001	<0.001
ADF	55.73 ^b^	38.42 ^c^	63.17 ^a^	61.85 ^a,b^	1.78	<0.001	<0.001	<0.001
Apparent total tract digestibility of ingredients, %								
GE	68.45	64.61	60.02	62.43	2.24	0.028	0.750	0.177
DM	66.37	63.33	64.08	67.04	2.29	0.702	0.985	0.121
OM	69.49	66.62	66.59	70.69	1.93	0.736	0.726	0.058
CP	78.41	68.98	58.56	55.25	3.86	<0.001	0.116	0.439
NDF	59.27 ^b^	50.16 ^c^	71.65 ^a^	75.19 ^a^	1.83	<0.001	0.131	0.002
ADF	50.66 ^b^	38.84 ^c^	62.32 ^a^	65.74 ^a^	2.27	<0.001	0.060	0.002

^1^ SEM, standard error of the mean; DE, digestible energy; ME, metabolizable energy; GE, gross energy; DM, dry matter; OM, organic matter; CP, crude protein; EE, ether extract; NDF, neutral detergent fiber; ADF, acid detergent fiber; ^a–c^ Means within a row without a common superscript letter differ (*p* < 0.05).

**Table 7:** Correlation coefficients among chemical characteristics and energy contents of 8 ingredients ^1, 2^ (dry matter basis).

Items	DEs	MEs	DEg	MEg	DEd	MEd	GE	Ash	OM	CP	EE	NDF	ADF
DEs	1	0.98 **	0.90 **	0.86 **	0.50	0.55	−0.06	−0.62	0.07	−0.33	0.24	−0.66	−0.73 *
MEs		1	0.86 **	0.85 **	0.56	0.58	−0.11	−0.72 *	0.15	−0.45	0.24	−0.62	−0.68
DEg			1	0.96 **	0.0.8	0.14	0.01	−0.43	−0.03	0.03	0.11	−0.77 *	−0.65
MEg				1	0.06	0.07	−0.13	−0.54	0.04	−0.11	0.05	−0.73 *	−0.56
DEd					1	0.98 **	−0.15	−0.57	0.23	−0.81 *	0.33	0.02	−0.37
MEd						1	−0.03	−0.53	0.23	−0.69	0.39	−0.06	−0.43
GE							1	0.38	−0.01	0.45	0.83 *	0.39	0.42
Ash								1	−0.63	0.77 *	−0.03	0.39	0.35
OM									1	−0.46	0.12	0.12	0.27
CP										1	−0.07	−0.10	0.11
EE											1	0.37	0.21
NDF												1	0.87 **
ADF													1

^1^ DEs, digestibility energy in sows; MEs, metabolizable energy in sows; DEg, digestible energy in growing pigs; MEg, metabolizable energy in growing pigs; DEd, difference between DEs and DEg; MEd, difference between MEs and MEg; GE, gross energy; DM, dry matter; OM, organic matter; CP, crude protein; EE, ether extract; NDF, neutral detergent fiber; ADF, acid detergent fiber. ^2^ Energy contents were expressed as MJ/kg and chemical compositions were expressed as %. * *p* < 0.05; ** *p* < 0.01.

**Table 8:** Prediction equations for digestible energy (DE), and metabolizable energy (ME) for sows in 8 ingredients ^1^ (dry matter basis).

Items	Regression Equations ^2^	Statistics				
		R^2^	RMES	AIC	BIC	*p*-Value
1	DEs = (1.05 × DEg) − (0.07 × CP) + 0.76	0.94	0.74	−2.63	−8.63	<0.001
2	DEs = (2.01 × DEg) − (1.03 × MEg) − (0.11 × CP) + 1.23	0.98	0.42	−11.61	−19.61	<0.001
3	MEs = (1.04 × DEg) − (0.09 × CP) + 0.52	0.96	0.64	−4.87	−10.87	<0.001
4	MEs = (1.59 × DEg) − (0.59 × MEg) − (0.11 × CP) + 0.73	0.97	0.59	−6.07	−14.07	0.002

^1^ DEs, digestible energy in sows; DEg, digestible energy in growing pigs; MEs, metabolizable energy in sows; MEg, metabolizable energy in growing pigs; CP, crude protein; RMSE, root mean square error; AIC, Akaike’s information criterion; BIC, Bayesian information criterion; ^2^ Values were determined with 6 observations per sample; chemical components were expressed as % dry matter; gross energy was expressed as MJ/kg dry matter.
